# Potential for Antigen-Specific Tolerizing Immunotherapy in Systematic Lupus Erythematosus

**DOI:** 10.3389/fimmu.2021.654701

**Published:** 2021-07-16

**Authors:** Sean Robinson, Ranjeny Thomas

**Affiliations:** ^1^ School of Medicine, Faculty of Medicine and Biomedical Sciences, University of Queensland, St Lucia, QLD, Australia; ^2^ University of Queensland Diamantina Institute, The University of Queensland, Princess Alexandra Hospital, Woolloongabba, QLD, Australia

**Keywords:** systemic lupus erythematosis, tolerance, dendritic cells, antigen (Ag), immunotherapies and vaccines

## Abstract

Systemic lupus erythematosus (SLE) is a chronic complex systemic autoimmune disease characterized by multiple autoantibodies and clinical manifestations, with the potential to affect nearly every organ. SLE treatments, including corticosteroids and immunosuppressive drugs, have greatly increased survival rates, but there is no curative therapy and SLE management is limited by drug complications and toxicities. There is an obvious clinical need for safe, effective SLE treatments. A promising treatment avenue is to restore immunological tolerance to reduce inflammatory clinical manifestations of SLE. Indeed, recent clinical trials of low-dose IL-2 supplementation in SLE patients showed that *in vivo* expansion of regulatory T cells (Treg cells) is associated with dramatic but transient improvement in SLE disease markers and clinical manifestations. However, the Treg cells that expanded were short-lived and unstable. Alternatively, antigen-specific tolerance (ASIT) approaches that establish long-lived immunological tolerance could be deployed in the context of SLE. In this review, we discuss the potential benefits and challenges of nanoparticle ASIT approaches to induce prolonged immunological tolerance in SLE.

## Introduction

Systemic Lupus Erythematosus (SLE) is a chronic autoimmune inflammatory disease that affects multiple organ systems. Clinical symptoms are heterogenous and range from mild to life threatening. SLE has a significant disease burden worldwide. Mortality in SLE has decreased significantly in the past 50 years (1), attributed to the use of immunosuppressive drugs, better supportive treatments and earlier diagnosis. Acute SLE-related mortality is usually due to uncontrolled inflammation and acute renal failure, while late mortality is linked to cardiovascular complications (2). Since the 1990’s late-phase clinical trials from more than 40 agents have failed in SLE. However, improvement in outcome measures, the efficacy of B cell activating factor (BAFF) and type 1 interferon (IFN) receptor 1 inhibition, and the promise of tolerance restoration, through drugs such as low-dose (LD) IL-2, underpin new optimism for future drug development (3–5). Tolerizing immunotherapies have the potential to revolutionize the treatment of autoimmune diseases by directly impacting adaptive immunity and restricting autoinflammatory responses by inducing peripheral immunological tolerance, either by expanding pre‐existing regulatory T cells (Treg) or by reprogramming autoreactive CD4+ T cells into Treg. While not extensively trialed in SLE yet, promising data in other autoimmune diseases provide learnings that may be applicable in SLE and patients at high-risk. In this review we examine the potential for antigen-specific immunotherapy to restore tolerance in lupus autoimmunity and discuss the advantages and challenges of immunotherapies and tolerizing approaches in SLE.

## Clinical and Etiological Considerations

SLE is 43.9% heritable, and the relative risk for siblings is 23.7. Shared environmental factors - such as infections - account for 25.8% of risk: the relative risk for spouses is 4.4 (6). Although the pathogenesis of SLE is not fully understood, the key elements are: dysregulated immune tolerance towards autologous nucleic acids with concurrent production of autoantibodies and autoreactive T-cells, disrupted clearance of apoptotic debris with increased self-antigen load and presentation to T cells, and interferon-driven inflammatory responses (7). Tissue damage – to skin, respiratory, renal, cardiovascular, central nervous and musculoskeletal systems – results from pathogenic autoantibodies, immune complex deposition and inflammation. SLE-associated environmental stressors, including UV light and infections may increase apoptotic load. With inadequate clearance, Toll Like Receptors (TLRs) recognize cellular debris (through damage associated molecular patterns, DAMPs) and initiate the inflammatory cascade, with pro inflammatory cytokine and type 1 interferon (IFN) production (8, 9). Presentation of nuclear self-antigens, such as dsDNA, chromatin, and RNA-containing antigens, to T and B cells induces the production of nuclear antigen-specific autoantibodies and autoreactive T-cells. There are multiple autoantibodies in SLE, including those directed towards nuclear antigen (ANA), double-stranded DNA (dsDNA), Smith (Sm), Ro, La, antiphospholipid (APL), and ribonucleoproteins (RNP) (10). Multiple lines of enquiry demonstrate loss of T and B cell tolerance in lupus. For example, the study of rare genetic variants associated with familial aggregation of lupus with other rheumatic autoimmune diseases identified regulation of T cell activation and T cell receptor (TCR) signaling as key underlying pathways (11). Furthermore, single cell transcriptomic analysis of peripheral blood (PB) identified antigen presenting cell, B cell and T cell dysregulation (12).

## Opportunities for Intervention With Tolerizing Approaches

SLE is classified (EULAR/ACR 2019 criteria) by the presence of ANA >1:80 and weighted scores for clinical and serological parameters (13). Some ANA+ individuals with very early disease or disease in evolution may fall below classification threshold. They may progress, to be re-classified as SLE, or may follow a milder and more stable clinical course. By the time of diagnosis, the majority of patients that meet SLE criteria will have some type of irreversible organ damage with clinical complications. The lupus disease course is characterized by flares and ongoing organ damage (14). Therapeutic intervention to a target of low disease activity (LLDAS) or clinical and serological remission reduces lupus-associated flares and organ damage, even when only achieved transiently (15). Typically, phase 3 trials of novel agents in SLE have struggled with small effect sizes due to disease heterogeneity, trial design issues, use of concomitant immunosuppression and endpoint validation (15). Instigation of trials in early disease and high-risk subjects not yet classified as SLE may improve the capacity to discriminate responses in patients with minimal organ damage. In a landmark phase 2 trial, a short course of T cell tolerizing immunotherapy Teplizumab, halved the progression of high-risk individuals to type 1 diabetes (16), while it had failed to meet its primary end-point in a phase 3 trial in recent-onset diabetes (17). Thus, T cell immunotherapy in people at risk (18) may be more effective before substantial organ damage.

SLE is associated with more autoantibodies than any other autoimmune disorder (19). Even before the development of disease pathology and symptoms, the pre-clinical phase is characterized by increased levels of autoantibodies, followed by a shift to multiple pathogenic autoantibodies associated with kidney, joint, heart, brain, skin and hematopoietic damage, including ANA, anti-dsDNA, anti-Sm, anti RNP, anti-APL, anti-Ro and anti-La (10). In general, anti-Ro, anti-La, and APL appear several years before the diagnosis of SLE, even in otherwise healthy individuals (20). In contrast, anti-dsDNA, anti-Sm, and anti–nuclear RNP antibodies usually appear only months before the clinical manifestations of SLE and are rarely present in healthy individuals (21, 22). In a retrospective study of 130 military personnel, use of hydroxychloroquine prior to SLE diagnosis delayed the onset of classified SLE and reduced the number of autoantibody specificities at and after diagnosis (23). At least 80% of individuals in this group met at least one SLE criterion prior to diagnosis. These results support a case for earlier therapeutic intervention with treatments of low toxicity before SLE classification and stratification of patients based on likelihood to respond. For example, current smoking was associated with elevated BAFF and reduced IL-10, particularly in ANA+ women (24). T cell expansion and type 1 IFN signatures were associated with a diagnosis of SLE in ANA+ individuals (25). Longitudinal cohort studies mapping the progression of SLE in auto-antibody positive healthy at-risk subjects will help identify early biomarkers of progression from autoantibodies to SLE, such as markers of functional loss of immune tolerance (26). Furthermore certain immune phenotypes might also be useful response biomarkers in mechanistic trials of immune tolerizing immunotherapies in individuals at high risk or with early disease.

Alternatively tolerizing approaches could be used to increase the likelihood that immunosuppressive drugs can be safely withdrawn without flare. In the BOLD clinical trial, standard immunosuppressive drugs were withdrawn and steroids substituted until flare, followed by reinstatement of standard therapy. During each phase cytokines and gene expression were analyzed to assess drug mechanism of action relative to baseline type 1 IFN transcriptomic signature. The authors identified that IL-17, IL-23 and BLyS pathways were changing with disease state and that IFN signature influenced the response of these pathways to individual drugs (27). This study provides an interesting proof-of-concept for a mechanistic trial of agents, such as tolerizing therapies, that could be introduced to reduce flare upon drug withdrawal. Although no cellular markers were included in this study, it demonstrates the utility of baseline IFN signature to stratify immune biomarker response outcomes. Future trials might also include Treg or T cell proliferation biomarkers. In this regard, a PB single cell transcriptomic resource shows co-clustering of a Treg T cell signature with dendritic cells (DC) lacking IFN-stimulated genes in lupus patients and healthy donors (12).

### Mechanisms of Immune Tolerance

Immunological tolerance is a vital aspect of a healthy immune system as it allows for appropriate immune responses to infectious and tumor antigens while containing potentially damaging immune responses to self-antigen and healthy tissue. Reviews of B and T cell antigen recognition and maturation can be found here (28, 29). During development, highly self-reactive T cells in the thymus are controlled by deletion (negative selection) of T cells with the highest affinity TCR for self-peptides, and by differentiation into CD4+ CD25+ FOXP3+ Treg cells (for non-deleted autoreactive CD4+ T cells), known as central tolerance. As negative selection depends on a TCR affinity threshold, weakly autoreactive T cells circulate in the periphery (30). Peripheral T cell tolerance mechanisms control autoreactive T cells through anergy (chronic antigen exposure deactivating T cell function), deletion, and regulation by Treg (derived from thymus or generated in the periphery). Antigen-specific Treg cells can suppress activation, proliferation and cytokine production of CD4+ T cells and CD8+ T cells through interaction with APCs, including B cells and dendritic cells (DCs), presenting cognate antigen. Functional antigen-specific peripheral Treg are key to restoration of immunological tolerance with immunotherapy as they can be induced from diverse T cell precursors, and their autoantigen specificity avoids generalized immune suppression (31).

Peripherally derived Treg cells, including IL-10+ type 1 regulatory T (Tr1) cells, are promising targets for immunotherapy to counteract established autoimmune diseases. Tr1 cells are induced in the periphery, predominantly from memory CD4 T cells, and are thus an important potential target for antigen-specific tolerance approaches (32–34). They are characterized by expression of IL-10, IFN-γ and TGF-β, lack of FOXP3 expression, expression of surface markers LAG3 and CD49b, and transcription factors EOMES and Tbet (35–37). With ongoing signaling by tolerogenic APCs presenting cognate peptide. Tr1 cells are long-lived, and associated with prolonged tolerance in multiple human autoimmune conditions (38–41).

DCs comprise a heterogeneous group of phagocytic APCs that sample soluble or apoptotic antigen at skin and mucosal surfaces, and process and present antigenic peptides to T cells in draining lymph nodes in context of MHC molecules. During an inflammatory episode, e.g. driven by infection, adjuvants, or damage, pathogen- or damage-associated molecular patterns (PAMPs, DAMPs) trigger the activation of the NF-κB pathway in DCs, enhancing their capacity to stimulate naïve T cells (42). DCs presenting antigens in the presence of regulatory signals that inhibit NF-κB, such as TGF-β or immunomodulatory drugs, skew antigen-specific T-cells towards regulation (43).

DC subsets developing from hematopoietic progenitors in bone marrow include plasmacytoid DC (pDCs), myeloid/conventional DC1 (cDC1) and myeloid/conventional DC2 (cDC2), based on surface markers and immune functions (44, 45). cDC1 and moDCs can cross-present antigens derived from tissues – including viral, tumor and self-antigens – to CD8 and CD4 T cells in context of MHC I and II (46, 47). cDC2 are potent activators of naïve T cells and induce CD4+ Th1, Th2, and Th17 responses (48, 49). pDCs produce high levels of type 1 IFN in response to nucleic acids *via* TLR7 and TLR9 signaling (50, 51). In SLE, pDCs produce high levels of type 1 IFN in response to nucleic acid and nuclear antigen (52). DCs are potential targets for immunotherapies to restore the dysregulated SLE immune system. For example, crosstalk between Treg cells and DCs through cell and cytokine signaling, controls DC activation and effector T cell activation. The signaling pathways for DC development and activation are crucial when considering drug cargo in the development of novel therapies in lupus.

### Pathogenesis of SLE

A basic understanding of the pathogenesis of SLE underpins a discussion on the development and effectiveness of novel immunotherapeutic agents. Here we highlight a few important factors that point to the underlying causes of SLE and that could be targeted in a therapeutic approach. For further reading please refer to the following reviews (53, 54).

SLE has been extensively studied using mouse models, which has helped illuminate pathogenesis. Some mouse models are genetically predisposed to the development of a lupus-like disease. Alternatively, a lupus-like disease may be induced in previously healthy mice. However, although spontaneous SLE models have been used to test potential therapeutics, successes in mouse models have not translated well in human trials. The NZB/NZWF1 (BW) mice and related strains develop spontaneous immune complex-mediated glomerulonephritis and mild vasculitis, with autoantibodies (anti-nuclear antibodies (ANA) and anti-dsDNA predominantly) (55). The MRL/*lpr* mouse is a unique spontaneous lupus mouse model that produces a variety of autoantibodies (ANA, anti-dsDNA, anti-Sm, anti-Ro and anti-La) and develops arthritis, cerebritis, dermatitis, vasculitis, and glomerulonephritis (56, 57). In induced mouse models of SLE, exogenous irritants or antigens are administered to replicate an environmental trigger (58, 59). Knock-out and knock-in mice backcrossed to lupus-susceptible backgrounds has expanded understanding of signaling cascades crucial for the development of SLE (60). Few antigens have been described in lupus mouse models, hampering the testing of antigen-specific approaches for lupus in general. However, antigen non-specific tolerizing approaches have been demonstrated to improve SLE disease manifestations in mouse models (61).

Hormones, smoking, ultraviolet light, and viral/bacterial infections are classic examples of exposures triggering SLE (62, 63). Estrogen and prolactin have been shown to drive immune responses underpin in part preponderance of women with SLE (64). Viral infection such as Epstein-Barr virus and cytomegalovirus have been suspected to play a triggering role in SLE pathogenesis whereas some pathogens have been linked to a protective role in SLE (65–67). Circulating levels of lipopolysaccharides have been shown to be elevated in SLE patients and to be correlated with disease severity, presumably through cytokine production (68, 69). Recently, alterations in gut microbiome have been linked to SLE disease status (70–72). This review will not thoroughly cover these environmental factors of but we note that they are important considerations when developing therapeutic trials for potential interventions.

#### Apoptotic Clearance, TLRs, Nucleic Acid Sensors and Cytokines

Abnormal apoptotic clearance can trigger TLRs and nucleic acid sensors on immune and non-immune cells and produce an immune response with cytokine production (7). Rare hereditary genetic mutations e.g. in *DNASE1L3* and *PRKCD* that lead to abnormal apoptotic pathways provide crucial insight into the role of apoptotic breakdown and debris clearance in SLE (73, 74). DNase I activity degrades chromatin in the apoptotic process and mice with a mutation in this enzyme had increased levels of anti-DNA antibody production (75). Smoking induces cellular damage and promotes cytokine production, and UV light enhances apoptotic turnover, and thus may increase self-antigen burden in susceptible individuals (76, 77)

Nucleic acid sensors are important surveyors of the environment and are specifically able to recognize viral infections and induce type I IFN production. Toll-Like receptors 3, 7, 8, and 9 shape the immune response by sensing cellular debris (78). In a pristane-induced lupus mouse model, TLR7, which senses single stranded RNA, was required for RNA-reactive autoantibodies (8). TLR9 senses unmethylated CpG sequence motifs. SLE patients with active disease have higher level of TLR9+ B cells and monocytes than healthy controls, and TLR9 levels correlated with antibodies to dsDNA (79, 80).

Type I and Type II IFN contribute a large role to the pathogenesis of SLE and become elevated prior to development of autoantibodies (81). Rare single gene disorders, grouped together as Aicardi-Goutiere’s syndrome, display gene defects that cause an overproduction of type I IFN (82). These patients display similar phenotypes to classic SLE, including autoantibodies.

There is a marked imbalance of T cell cytokines in SLE, with low levels of IL-2 accompanied by elevated IL-17 and IL-6 (83). IL-2 is a key cytokine in Treg development, survival and maintenance. It restricts Th17 cell development (84, 85). Elevated levels of IL-17 are thought to induce tissue inflammation and recruitment of immune cells. B cell activating factor (BAFF or BLyS), expressed by stromal and immune cells, promotes B cell activation in SLE and its levels positively correlate with antibody levels (86, 87).

#### Loss of Immune Tolerance

The process of autoimmune disease development can be roughly categorized into three stages: 1) a priming phase that includes an inciting event or accumulation of events in individuals at genetic and environmental risk; 2) the onset of clinical symptoms marked by organ-specific inflammation; and 3) a chronic inflammatory tissue-destructive phase (88). During the transition to clinically significant symptoms, regulatory processes, including Treg cells, fail to control pathological autoreactive B and T cells. This imbalance perpetuates the processes of bystander activation, epitope spreading and uncontrolled cytokine and antibody production. Epitope spreading involves the diversification of epitope specificity from the initial dominant epitope-specific immune response (89). The specificity of the autoimmune response spreads to include additional self-epitopes besides the initiating self-antigens. Chronic inflammation promotes tissue damage and cascading self-antigen presentation, expanding autoreactive T-cell specificities, including cryptic or sequestered epitopes (90). For example, late-stage SLE is characterized by an explosion of autoantibodies, apparently the result of chronic inflammation and epitope spreading (19). Bystander activation occurs with stress, infection or trauma-induced activation of tissue APCs, activating T cells of additional specificities, which further promote inflammation and tissue damage. Bystander T cells can provide help to B cells for autoantibody production, or to cross-presenting DCs presenting tissue-derived self-antigen (91). Treg cells may control bystander T cells and epitope spreading through interaction with cross-presenting DCs. In a rheumatoid arthritis mouse model Treg cell depletion promoted the expansion of pathogenic autoreactive T cells, an increase in inflammatory cytokines, and B‐cell epitope spreading (92).

SLE is marked by abnormal B and T cell interactions and spontaneous germinal centers in secondary lymphoid organs (93–95). In SLE there is loss of functional Treg and induction of effector T cells that produce proinflammatory cytokines and BAFF, which is not normally observed in healthy people (96, 97) (98). Multiple lines of evidence demonstrate the importance of Treg in lupus pre-clinical models. In the NZB/NZWF1 spontaneous model, Treg cell adoptive transfer delayed SLE progression, reduced renal pathology, and improved survival (61), while Treg depletion accelerated disease development (99). In human SLE, most but not all studies demonstrate a reduced frequency of Treg cells (100, 101). Targeted depletion of pDCs decreased SLE-associated glomerulonephritis in mice (102, 103). In human SLE, while pDC are decreased in the blood, they are increased in lupus-affected organs, suggesting their chemo-attraction and possible expansion at these sites (104–106).

## Immunological Tolerance Therapeutics in SLE

### Current Tolerizing Strategies for SLE

There are multiple potential targeted immunotherapies undergoing research and development and early phase clinical trials for SLE (107, 108). Most techniques exploit antigen‐presentation pathways of APCs or attempt to deliver antigenic cargos to locations thought to be involved in regulatory T‐cell formation (109). Other strategies target antigen‐specific T‐cells to re‐program pathogenic autoreactivity into disease‐suppressing autoregulation (110, 111). **Table 1** outlines some promising therapeutic directions aiming to enhance immune tolerance by targeting DCs and Treg cells.

**Table 1:** Treg and DC based Therapies without Autoantigen.

Therapy	Mechanism	Clinical trial for SLE	References
Adoptive Treg cell or DC transfer	Non antigen-specific increase Treg cells, Antigen-specific tolerogenic DC immunotherapy to induce Treg cells	Yes for Tregs, No for DCs	(112–116)
HSCT/MSCT	Non antigen-specific immune tolerance	Yes	(117, 118)
Low-dose IL-2	Non antigen-specific increased survival, proliferation and/or function of Treg cells	Yes	(3)
Targeted DC immunotherapy	Induce tolerance through tolerogenic antigen delivery to DCs	No	(119, 120)

#### Expanded Treg Cell Transfer

Several groups have developed methods to expand Treg cells *ex vivo* for reintroduction as an autologous cell therapy product. Treg cells can be isolated from peripheral blood or umbilical cord blood, but must be expanded due to their low frequency. In vitro expansion strategies include anti-CD3/CD28-coated beads, with addition of IL-2 and/or TGF-β and rapamycin (121). Proof of concept experiments in lupus-prone mice showed that *ex vivo*-expanded Treg cells suppressed glomerulonephritis and prolonged survival (61, 122). *Ex vivo*-expansion of Treg cells in the presence of immunosuppressive drugs or Treg transfer into patients on immunosuppressants can be challenging, as the drugs may hinder expansion or change function (112). Furthermore, the process requires a good manufacturing practice (GMP) environment, which is challenging and expensive. A clinical trial using *ex vivo*-expanded autologous polyclonal Treg cells in patients with autoimmune disease was terminated in November 2019 due to screen failures and low enrolment. In a case report, the treatment was shown to be safe and clinical disease activity to be stable in a single SLE patient. Infused labeled Treg cells were transiently observed in PB then in diseased SLE skin, accompanied by skewing from Th1 to Th17 immunity locally (123). Treg are highly plastic and may differentiate to Th17 in inflammatory settings and where IL-2 is limiting (124). Larger studies are needed to understand the impact of Treg therapy on disease severity.

#### HSCT/MSCT

Hematopoietic and/or mesenchymal stem cell transfer (HSCT and MSCT, respectively) have been trialed in patients with severe autoimmune diseases, including SLE, who have failed standard therapy. In SLE patients, HSCT has successfully induced long-term remission (125). In 15 patients with severe SLE evaluated up to 8 years after HSCT, CD4^+^CD25^high^Foxp3^+^ Treg and LAP^high^TGF-β^+^CD8^+^Foxp3^+^ cells were restored to levels and function similar to healthy subjects (117). These promising results suggest that HSCT may reestablish immune tolerance by replenishing multiple types of Treg cells. However, as HSCT is associated with significant risks, treatment complications and cost, it is currently reserved for treatment-refractory patients. A 4-year follow-up of an open-label trial of MSCT in 87 treatment-refractory SLE patients found a 28% remission rate post-infusion (118). While double-blind placebo-controlled trials are needed to understand the true benefits of MSCT, these trials provide evidence that tolerance may be successfully re-established in SLE.

#### Low-Dose IL-2

IL-2 levels and CD25 expression by Treg are reduced in SLE patients and murine lupus models (126–128). IL-2 plays a pleiomorphic role in the immune system. One of its functions is to expand and promote survival of Treg cells (129, 130). Reduced IL-2 favors the differentiation of IFN-γ-producing Th1 and IL-17 producing Th17 cells and their accumulation in skin and kidneys (131, 132), and is associated with inflammation. In lupus-prone mice, IL-2 treatment increased levels of Treg cells in lymphoid and peripheral organs and protected them from SLE-related organ damage (99, 133). There have been several trials in lupus showing safety and Treg expansion (128, 134). In a recent double-blind placebo-controlled clinical trial in patients with suboptimally controlled SLE, LD IL-2 for 12 weeks (s.c. alternate days for three 2-week cycles), the SLE Responder Index (SRI)-4 response rates at week 12 were 55.17% and 30.00% in LD IL-2 and placebo groups respectively (p=0.052). Although the primary end point was not met, the significantly greater lupus nephritis complete remission rate in the LD IL-2 arm was notable. Immunologically, IL-2 supplementation significantly increased Tregs and NK cells but did not change total CD4+ or CD8+ T cells and there was no increase in viral load of pre-existing viruses (3). While promising, LD IL-2 dosing may be complicated by concomitant expansion of regulatory and cytotoxic cells. Furthermore, development of neutralizing autoantibodies with continued treatment is a potential risk (135). Targeted IL-2 therapies may allow more precise manipulation of the immune response and longer duration of action. For example, anti-CD4 and anti-CD2-coated poly(lactic-co-glycolic) acid (PLGA) nanoparticles loaded with IL-2 and TGFβ expanded Treg cells *in vitro* and *in vivo* in the BDF1 lupus pre-clinical model (136). In a recent phase 1b clinical trial of a polyethylene glycol (PEG) conjugate of IL-2 (NKTR-358) in patients with mild to moderate SLE, dose-dependent increases in Tregs (up to 11 fold) were observed, which returned to baseline 20-30 days post-dose (137). Anti-IL-2 antibodies were not reported.

#### Tolerogenic DCs

DCs play a critical role in maintaining self-tolerance. Indeed, targeting steady-state skin migratory DC with antigen coupled to DC-selective antibodies induced antigen-specific tolerance (138). Tolerogenic DCs can also be generated *in vitro* from monocytes or murine bone marrow precursors in the presence of NF-κB inhibitors 1,25 (OH)_2_ vitamin D3 (calcitriol), rapamycin or glucocorticoids. After proof-of-concept studies in experimental animal models (139, 140), several groups translated antigen-specific immunotherapy using modified or tolerogenic autologous DCs and autoantigenic peptides to clinical trials for MS (113) and RA (114, 115). These trials demonstrate the feasibility and safety of this approach, with preliminary evidence of an immunomodulatory effect in RA. In two pre-clinical lupus models, histone antigen-loaded tolerogenic DCs improved clinical scores, increased Treg in affected skin and reduced anti-histone autoantibodies (141). Tolerogenic DCs exposed to apoptotic cells were generated from PB monocytes derived from lupus patients (142). Other approaches have been developed to target DCs directly *in situ*, including a PLGA nanogel to deliver the immunomodulator mycophenolic acid (MPA) to DCs (119, 120). DCs took up the PLGA-lipid-MPA nanogel more efficiently and with better DC suppression than a PLGA nanogel. In a murine lupus model, PLGA-MPA nanogel increased median survival by 3 months when given prophylactically and by 2 months when given to mice with advanced renal damage. Consistent with the local effects of MPA on DCs, treated mice had a substantial reduction in DC-derived inflammatory cytokines such as IFN-γ and IL-12. Although not strictly immune tolerance, this approach achieves sustained delivery of MPA to induce a prolonged anti-inflammatory effect.

#### Lupuzor

Lupuzor (rigerimod or IPP-201101) is a 21aa peptide representing residues 131–151 of the 70K spliceosomal protein within the U1 small nuclear RNP, phosphorylated at Ser140. This promiscuous peptide sequence was identified using *ex vivo* peptide screening techniques (143). This epitope is recognized by IgG antibodies and CD4+ T cells from H‐2^k^ MRL/*lpr* and H‐2^d/z^ (NZB × NZW)F1 lupus‐prone mice (143, 144). With i.v. delivery, the peptide inhibits chaperone-mediated autophagy and reduces B cell MHC class II expression (145). Two trials of IPP-201101 immunotherapy in SLE demonstrated safety and potential efficacy (146, 147). However, IPP-201101 failed to meet its primary end point of superiority over standard care in phase III clinical trials (148). The peptide seemed to have non antigen-specific immunomodulatory properties, rather than inducing antigen-specific regulation, and this may be why it was not superior to standard care. Standard of care high dose glucocorticoids and immunosuppressive drugs are likely more bioavailable than an immunosuppressive peptide.

These treatment strategies are antigen non-specific and use nanoparticles (NP) to deliver biologics or immunosuppressive drugs. In the following sections we consider antigen-specific tolerizing approaches using NP in SLE.

### Potential Antigen-Specific Tolerizing Platforms for SLE

Antigen-specific therapies for autoimmune diseases involve the delivery of autoantigen in a regulatory context, with or without a delivery vehicle that reprograms APCs by modulating NF-κB, or by antigen delivery to a naturally tolerogenic site e.g. by targeting steady-state skin-draining APCs or the liver tolerogenic environment. Some approaches may directly differentiate Tr1 cells from memory T cells.

Peptide alone, delivered s.c., can be tolerogenic. For example, an islet proinsulin epitope returned promising results in phase 1 trials in T1D (149). Peptides that associate with MHC class II molecules expressed by APCs, without the need for antigen processing, can directly target steady-state DC *in vivo*. Such antigen processing independent epitopes (“apitopes”) selectively bind steady-state DCs *in vivo* because steady-state DCs bear peptide receptive/empty MHC II at the cell surface, which is lost upon DC activation (150, 151). Apitopes induce tolerance through induction of anergy and generation of Tr1 cells (152). Tr1 cells selectively express a tolerance-associated set of genes (153, 154). Phase 1 and 2 clinical trials of multiple low dose apitope delivery have been undertaken in Graves’ disease and MS respectively. While low-dose soluble antigen administered s.c. is non-immunogenic, high dose peptide, aggregates or protein complexes can induce an immune response through immune complex formation, macrophage or DC activation and development of autoantibodies.

#### NPs Delivering Antigens and Immunomodulators

Liposome formulations loaded with peptide or protein antigens and various NF-κB inhibitors, including curcumin, quecertin and BAY11-7082 induced antigen-specific tolerance in mice with antigen-induced arthritis (155). We also developed and undertook pre-clinical studies of liposomes co-encapsulating calcitriol and peptide. Calcitriol/peptide liposomes promoted the differentiation of antigen-specific Foxp3^+^ Treg, anergy of Tmem, and IL-10 production upon restimulation with antigen *ex vivo* (156). Notably, liposomes were preferentially taken up by activated PD-L1^+^ migratory DCs, and regulation was PD-L1-dependent. We translated this to a phase 1b clinical trial in RA. Other groups have co-encapsulated antigens in NPs with either rapamycin (157) or aryl hydrocarbon receptor (AhR) ligands (158) for *in vivo* uptake by DC. With substitution of suitable lupus antigenic peptides, these liposome or NP approaches could be adapted to lupus patients.

#### Nanoparticles Leveraging Natural Tolerogenic Processes

Other research groups have developed NPs that resemble apoptotic bodies, to promote a tolerogenic response to encapsulated antigen. Specifically, i.v. administration of 500nm PLGA particles encapsulating antigen induced antigen specific tolerance (159, 160). These relatively large, negatively-charged particles are preferentially taken up by DCs and macrophages expressing MARCO, and induce antigen-specific suppression in the absence of an immunomodulatory drug (161). Another strategy to mimic signals from apoptotic bodies uses phosphatidylserine (PS) liposomes. During apoptosis, the PS phospholipid translocates from the inner leaflet to the outer leaflet of the lipid bilayer of the dying cell. PS liposomes suppressed pre-clinical models of T1D and acute EAE in a non-antigen-specific manner (162, 163). It is unclear whether this technique would succeed in SLE, which is characterized by impaired clearance of apoptotic cells.

#### Peptide-MHC NPs

The TCR may also be directly targeted with NPs coated with peptide loaded onto MHC class I or II, without co-stimulation. After i.v. delivery of iron oxide nanoparticles coated with peptide-MHC class I complexes (pMHC-I) they suppressed autoreactive CD8+ memory T cells and converted them to a regulatory, anergic phenotype (110). Nanoparticles coated with pMHC-II differentiated cognate autoreactive CD4 memory T cells into Tr1 cells producing IL-10 (111, 164). Nanoparticles coated with pMHC-II suppressed autoimmune symptoms in several pre-clinical models in an antigen-specific manner, without compromising systemic immunity (111). To date, this approach has not been translated to clinical trials.

**Figure 1:**
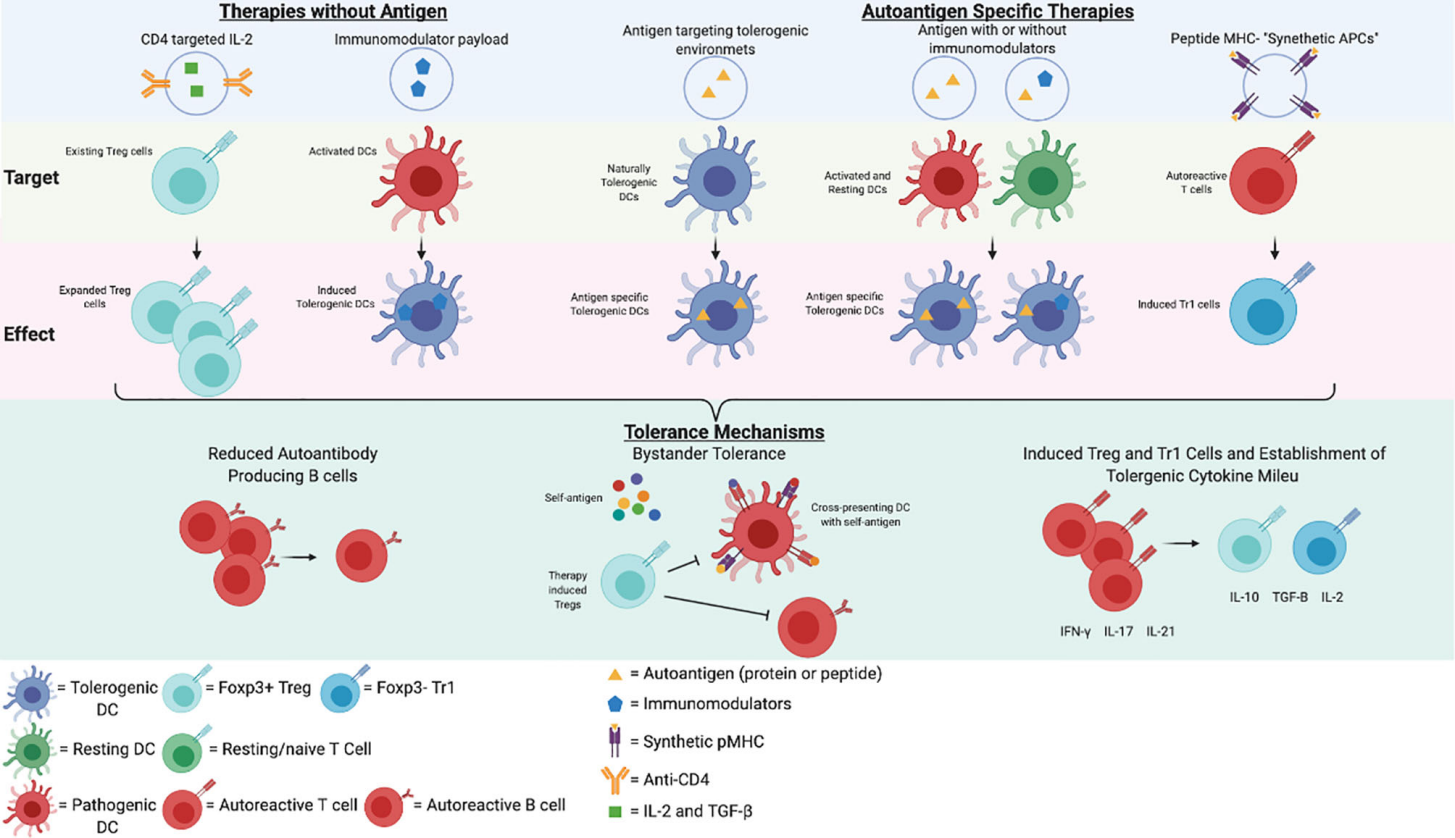
Potential Nanoparticle Therapies in SLE. A representation of potential immunotherapies for SLE and a simplified schematic of their mechanisms of action. This figure was created in BioRender.com.

Thus, a wide array of nanoparticle technologies has been developed. **Figure 1** describes some of the technologies incorporating autoantigens, immunomodulatory drugs, or targeting strategies, or a combination of strategies. In summary, approaches that promote the expansion of antigen-specific Treg cells, particularly Tr1 cells derived from autoreactive memory T cells, will be required to control bystander cytokine production and epitope spreading in multi-system autoimmune diseases, such as SLE.

## Autoantigens in SLE

Many autoantigens potentially contribute to the development of SLE and it is unclear which antigen(s) should be targeted in antigen-specific immunotherapy. Several promiscuous epitopes have been described across mice and humans. Choosing an antigen is challenging because there are many different pre-clinical lupus models, the disease is highly heterogeneous in humans, and translation of antigen discovery from mouse to human is difficult. However, assays of T cell responses in organ-dominant lupus “endotypes” may offer opportunities to identify relevant skin, joint, renal, neurological and hematologic antigenic epitopes that are suitable for clinical trials with focused outcomes.

Despite these hurdles, antigen-specific immunotherapies with a single strong autoantigen that also promote bystander tolerance could leverage the expansion of antigen-specific Treg cells and the suppression of cross-presenting DCs carrying relevant epitopes from diseased tissue to draining lymph nodes. Bystander tolerance has been demonstrated for several immunotherapies in pre-clinical models, including apitopes, peptide/calcitriol liposomes, and pMHC-NP, associated with the modulation of immune responses other than the epitope included in the immunotherapy (111, 165). For example, in a type 1 diabetes mouse model, calcitriol liposomes encapsulating a single islet CD4 epitope suppressed the disease and bystander islet-reactive CD8+ cytotoxic T cells (166). The advantages of harnessing bystander tolerance mediated by Treg cells, compared to generalized immunosuppression, is that bystander suppression is tissue-restricted, and Treg develop from autoreactive memory T cells. However, suitable antigenic epitopes must be identified.

Haplotypes containing DR2/DQ6, and DR3/DQ2 alleles are associated with SLE (167). DR2/DR3 heterozygosity is associated with anti-Ro, anti-La, anti-Sm, anti-ribosomal-P or anti-ribonuclear protein antibodies, while HLA-DR homozyosity is associated with anti-Sm and anti-dsDNA (167). HLA-restriction poses a potential hurdle for the applicability of peptide-specific immunotherapies, as peptides need to be identified and matched to patient MHC class II. Long antigenic sequences or mixtures of epitopes that cover a large percentage of the diseased population provide potential solutions. HLA-restricted soluble or NP-associated peptide immunotherapy may be a good way to achieve some early positive immune outcomes of antigen-specific immunotherapies, including bystander tolerance in proof-of-concept clinical trials. Subsequently, tolerizing immunotherapies with multiple autoantigens or proteins could be further tested.

Strategies to identify potential self-peptides include: screening autoreactive T cell proliferation or cytokine production *ex vivo*, peptide elution from MHC II molecules, and autoantibody binding epitopes. Immunization studies in DR3 transgenic mice have been used to map DR3-restricted SmD T cell epitopes (168). Studies investigating apoptotic cell-derived self-epitopes recognized by pathogenic T cells in human and lupus-prone mouse models identified potential histone epitopes, including histone H1’_22-42_, H4_16–39_, H4_71–94_ and H3_82–105_ (169, 170). These extended epitopes bind multiple HLA-DR allomorphs. Most also bound anti-histone autoantibodies (171, 172). In human PB cultures, these peptides promoted TGF-β secretion and expanded Foxp3+Treg cells in the presence of IL-2 *in vitro (* 170). In SVF1 lupus-prone mice, s.c. administration of H4_71–94_ every 2 weeks induced TGF-β-producing pDCs and Treg cells and protected mice from renal disease (173, 174). A 70K-U1RNP_131-151_ T helper epitope was identified in NZBxNZW F1 and MRL/Fas(lpr) mice, which led to further identification of SmD1 and hnRNP A2/B1 epitopes in each strain. Of interest the SmD_95-119_ epitope recognized by anti-Sm antibodies is homologous to an Epstein-Barr virus EBNA I peptide, suggesting a mechanism for epitope spreading through bystander T helper cells (144, 175). Certain nuclear antigens tend to induce epitope spreading to related other nuclear antigens in mouse models (**Table 2**). If administered as antigen-specific tolerizing immunotherapy, one would therefore predict induction of bystander tolerance (183).

**Table 2:** Epitope spreading in mouse models after autoantigen immunization.

Antigen	Autoimmune Epitope Spread	Reference
Ro 60 (aa 316–335)	Ro60, La, Sm, U1RNP	(176)
SmD1 protein	A-RNP, SmD	(176)
SmB protein	A-RNP, SmD	(176)
SmD183–119	SmD, dsDNA	(177)
SmB′/B aa PPPGMRPP	SmD, 70k-/A-U1RNP	(178)
Murine La (aa 13–30)	Ro52	(179)
A2/B1 hnRNP (aa 50–70)	hnRNP	(180)
Nucleosome (lupus-prone mice)	dsDNA, nucleosome, histone	(181)
La (aa 13–30)	La, Ro	(179)
Histone H1	H2, ssDNA	(182)

Further research into SLE immunotherapy would benefit greatly from a humanized model that could better represent the human immune system (184, 185).

## Conclusion

SLE is a devastating autoimmune disease with a large unmet need for better therapies. Promising work has identified some immunological markers of immune tolerance in individuals at risk who have not progressed to a diagnosis of SLE, and some nuclear-derived antigenic epitopes that may be presented by multiple MHC II molecules. More work is needed to carefully map the autoantigen specificity and HLA restriction of expanded T cells in patients with recent-onset SLE. The pre-clinical phase and milder organ-specific endotypes of SLE provide potential opportunities to intervene in individuals with a less aggressive or more focused disease processes, associated with lower levels of organ damage. Technological platforms showing promise in early-phase clinical trials or preclinical models in other autoimmune diseases could be adapted for trials in SLE. Given the clinical complexity, sensible beginning strategies would comprise small mechanistic studies with immune biomarker and safety outcomes in well-defined limited disease settings.

## Author Contributions

SR drafted and edited manuscript, and compiled figure. RT edited manuscript, figure and tables. All authors contributed to the article and approved the submitted version.

## Funding

This manuscript is part of a project that has received funding from the Innovative Medicines Initiative 2 Joint Undertaking under grant agreement No 777357. This Joint Undertaking receives support from the European Union’s Horizon 2020 research and innovation programme and EFPIA. RT was supported by NHMRC grant 1079238 and by Arthritis Queensland.

## Conflict of Interest

The authors declare that the research was conducted in the absence of any commercial or financial relationships that could be construed as a potential conflict of interest.
